# To Correspondents

**Published:** 1893-10

**Authors:** 


### TO CORRESPONDENTS.

The necessity of giving a portion of the selected papers read at the Columbian Dental Congress has forced the enlargement of this number, and has obliged us to defer much interesting matter until the next issue. We ask the indulgence of our regular contributors.

